# Inhibition of Epstein-Barr Virus Lytic Cycle by an Ethyl Acetate Subfraction Separated from *Polygonum cuspidatum* Root and Its Major Component, Emodin

**DOI:** 10.3390/molecules19011258

**Published:** 2014-01-20

**Authors:** Ching-Yi Yiu, Shih-Ying Chen, Tsai-Hsiu Yang, Che-Jung Chang, Dong-Bor Yeh, Yi-Jie Chen, Tsuey-Pin Lin

**Affiliations:** 1 Department of Otolaryngology, Chi Mei Medical Center, Liouying, Tainan 717, Taiwan; 2 Department of Health and Nutrition, Chia-Nan University of Pharmacy and Science, No.60, Sec. 1, Erren Rd., Rende Dist., Tainan 717, Taiwan; 3 Department of Biotechnology, Chia-Nan University of Pharmacy and Science, Tainan 717, Taiwan

**Keywords:** *Polygonum cuspidatum*, emodin, antiviral activity, EBV

## Abstract

*Polygonum cuspidatum* is widely used as a medicinal herb in Asia. In this study, we examined the ethyl acetate subfraction F3 obtained from *P. cuspidatum* root and its major component, emodin, for their capacity to inhibit the Epstein-Barr virus (EBV) lytic cycle. The cell viability was determined by the MTT [3-(4,5-dimethyldiazol-2-yl)-2,5-diphenyltetrazolium bromide] method. The expression of EBV lytic proteins was analyzed by immunoblot, indirect immunofluorescence and flow cytometric assays. Real-time quantitative PCR was used to assess the EBV DNA replication and the transcription of lytic genes, including BRLF1 and BZLF1. Results showed that the F3 and its major component emodin inhibit the transcription of EBV immediate early genes, the expression of EBV lytic proteins, including Rta, Zta, and EA-D and reduces EBV DNA replication, showing that F3 and emodin are potentially useful as an anti-EBV drug.

## 1. Introduction

Epstein-Barr virus (EBV) is an oncogenic human herpesvirus, which infects lymphoid cells and epithelial cells [1]. Although the infection is often asymptomatic, it causes infectious mononucleosis and is associated with a number of human cancers, including Burkitt’s lymphoma [2], nasopharyngeal carcinoma (NPC) [3], Hodgkin’s disease [4] and gastric carcinoma [5]. There are two stages of the virus life cycle, the latent and lytic cycles. The reactivation of EBV from latency to the lytic cycle is necessary for the virus to produce virions and establish infections [6,7]. At onset of the lytic cycle, EBV expresses two transcription factors, Rta and Zta, which are transcribed from BRLF1 and BZLF1, respectively [8]. These two proteins trigger an ordered cascade of the expression of viral lytic genes, including that of BMRF1 and BALF5, which encoded diffused early antigen (EA-D) and DNA polymerase [9]. After EBV DNA replication, late genes, such as those encoding virus capsid antigens and membrane proteins, are expressed, followed by the production mature virions [10]. Some studies have suggested that the EBV lytic cycle causes tumorigenesis by the induction of cytokines [11]. Moreover, EBV lytic proteins have been shown to contribute to human pathology, such as Zta, suggested to induce the production of several oncogenic and inflammatory cytokines production [12,13]. Therefore, an effective strategy to block the viral lytic cycle is of value to prevent or treat EBV-associated disease and to improve the clinical outcome. The nucleoside analogs, such as acyclovir and ganciclovir, are frequently used to treat EBV infection, and have reported to be effective in infectious mononucleosis [14], post-transplant lymphoproliferative disorder [15], HIV-relative lymphoma and hairy leukoplakia [16]. Earlier studies have established that lytic EBV replication is inhibited by acyclovir and ganciclovir, which inhibit specifically the function of viral-encoded DNA polymerase [17], but not the expression of immediate-early or early lytic proteins. In addition to chemicals, epigallocatechin gallate (EGCG) [18], resveratrol [19], ethanolic extract from *Andrographis paniculata* and andrograpolide [20] are also suggested to be effective in inhibiting the EBV lytic cycle through suppressing the expression of the EBV immediate-early genes transcription and the expression of lytic proteins, including Rta, Zta and EA-D.

*Polygonum cuspidatum* is a herbal medicine commonly used for the treatment of atherosclerosis, as well as other medical ailments including cancers, asthma, hypertension and coughs [21]. The methanolic extract of *P. cuspidatum* root contains anthraglycoside B, physcion, piceid, emodin and resveratrol [22]**.** Previous studies showed that the ethanolic extract of *P. cuspidatum* inhibits hepatitis B virus replication [23] and EBV lytic cycle [24]. However, no study has reported on the inhibition of EBV immediate-early gene expression and DNA replication by emodin. Thus, the purpose of our study was to evaluate the ethyl acetate emodin-containing subfraction F3 separated from *P. cuspidatum* root and its major component, emodin, on the inhibition of the transcription of EBV immediate early genes, the expression of EBV lytic proteins, including Rta, Zta, and EA-D and DNA replication.

## 2. Results and Discussion

### 2.1. Analysis of Components in the Ethyl Acetate Subfraction F3 Isolated from Polygonum cuspidatum

**Figure 1:**
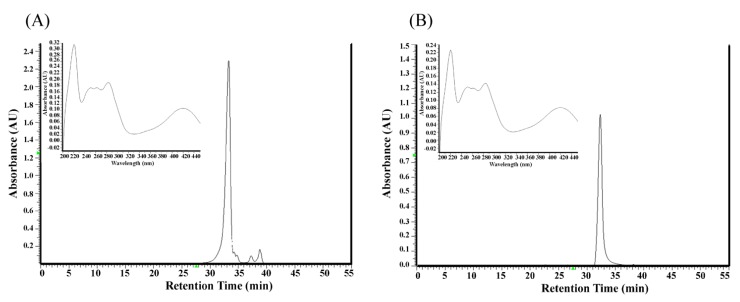
HPLC chromatograms and UV spectra of the ethylacetate subfraction F3 isolated from *Polygonum cuspidatum root* (**A**) and pure standard of emodin (**B**). Mobile phase: methanol–water (methanol: 0–15 min, 30%–50%; 15–35 min, 50%–90%; 35–45 min, 95%); flow rate: 1.0 mL/min; detection wavelength: 280 nm.

The ethyl acetate subfraction F3 was obtained from the ethanolic extract of *Polygonum cuspidatum* root through partition with hexane and ethyl acetate and separation by semi-preparative high pressure liquid chromatography. Its components were analyzed by high liquid pressure chromatography and the identification of target compounds in F3 was based on their retention time and UV spectra, compared against a pure standard of emodin. We found that the peak eluting at 33.01 min could be ascribed to emodin (Figure 1). The amount of emodin in F3 was 68.2%.

### 2.2. Effect of F3 and Emodin on the Viability of P3HR1 Cells

**Figure 2:**
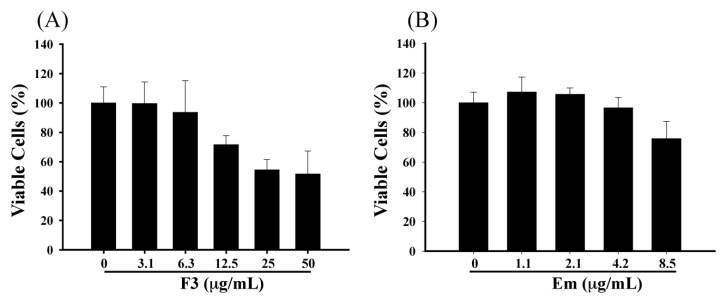
Effects of F3 and emodin on cell viability. P3HR1 cells (1 × 10^5^ cells/mL) were treated with F3 (**A**) or emodin (Em) (**B**). Cell numbers and viability were monitored by MTT assay after 24 h of treatment.

To address the question of whether F3 and emodin can inhibit the EBV lytic cycle, first, we sought to determine the cytotoxicity of F3 and emodin to P3HR1 cells. F3 or emodin were added to P3HR1 cells (1 × 10^5^ cells/mL), and after 24 h treatment, the cell viability was determined by the MTT method. Results showed that F3 and emodin slightly affected the viability of P3HR1 cells. When cells treated with 12.5 μg/mL of F3 and 8.5 μg/mL of emodin, the cell viability showed a significant decrease by 28.4% and 24.3%, respectively (Figure 2). However, this study indicated that F3 and emodin did not influence the cell viability at concentrations below 6.3 μg/mL and 4.2 μg/mL, respectively (Figure 2).

### 2.3. Inhibition of EBV Lytic Proteins Expression by F3

**Figure 3:**
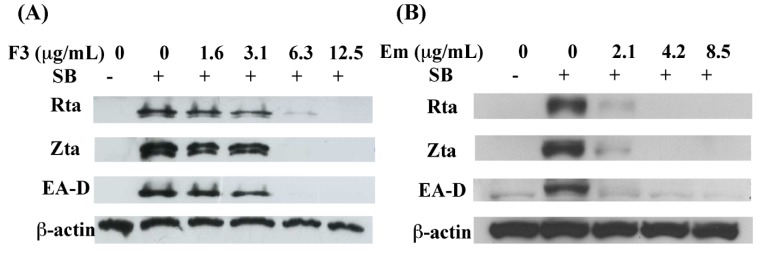
Inhibitory effects on the expression of EBV lytic proteins, including Rta, Zta and EA-D by F3 and emodin. The F3 (**A**) or emodin (Em) (**B**) was added to P3HR1 cells induced by sodium butyrate (SB). Syntheses of Rta, Zta and EA-D by EBV were studied by immunoblot analysis at 24 h after lytic induction with anti-Rta, anti-Zta, anti-EA-D and anti-β-actin antibodies.

The immediate early proteins, Rta and Zta transcription factors, are involved in lytic cycle, where they activate the expression of numerous early and late proteins, such as BMRF1 gene, EA-D. EA-D enhances the effectiveness of viral DNA polymerase and promotes EBV DNA replication, with the subsequent release of infectious virions. The detection of lytic proteins is a useful tool to examine the status of EBV lytic reactivation. The purpose of this study is to explore F3 and emodin whether inhibited the expression of EBV lytic proteins. P3HR1cells (3 × 10^6^) were seeded for 24 h then treated with various concentration of F3 or emodin before sodium butyrate. After 24 h, cell extracts were harvested and the EBV lytic proteins, Rta, Zta and EA-D were analyzed by immunoblot analysis. These results showed that F3 and emodin significantly inhibited the expression of Rta, Zta and EA-D at the concentration of 3.1 μg/mL and 2.1 μg/mL, respectively. At 6.3 μg/mL of F3 and 4.2 μg/mL of emodin completely inhibited the expression of Rta, Zta and EA-D (Figure 3).

### 2.4. Indirect Immunofluorescence Analysis of EBV Lytic Proteins Expression

**Figure 4:**
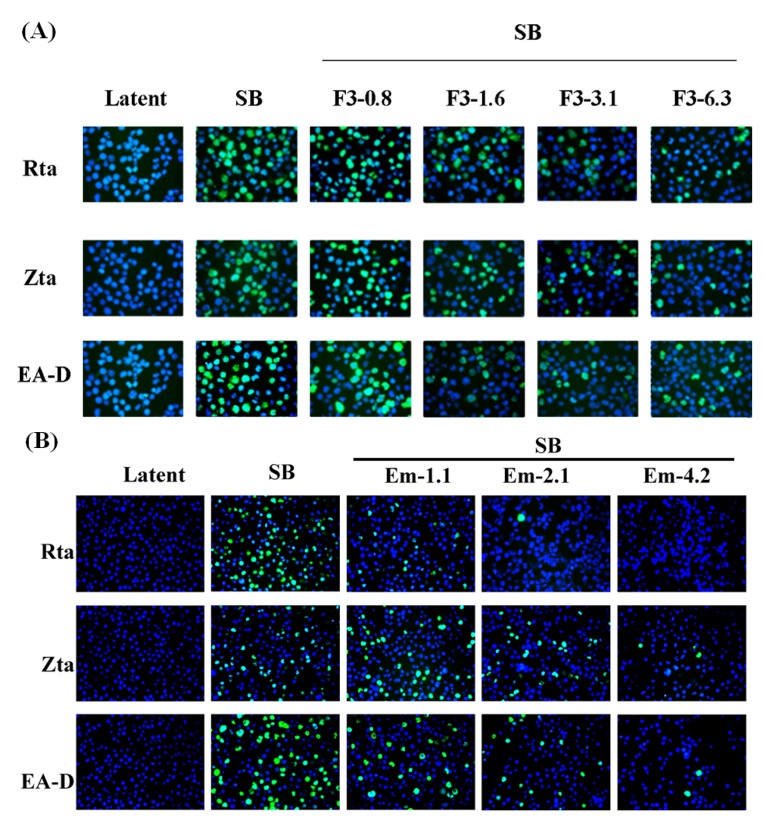
Indirect immunofluorescence analysis of inhibition of the expressions of EBV lytic genes by F3 and emodin. P3HR1 cells were pre-treated with F3 (0.8, 1.6, 3.1 and 6.3 μg/mL) (**A**) or emodin (1.1, 2.1 and 4.2 μg/mL) (**B**) before lytic induction for 24 h. Cells were then harvested and the expressions of the lytic EBV proteins were assessed by immunofluorescence. P3HR1 cells untreated with sodium butyrate (SB), F3 and emodin were used as a negative control (Latent). The following primary antibodies were used: mouse monoclonal anti-Rta, anti-Zta, and anti-EA-D antibodies. The secondary antibodies were used: Alexa Fluor 488-conjugated goat anti-mouse IgG. DNA was visualized by DAPI staining; cells were examined under a fluorescence microscope.

To further examine the inhibitory effects of F3 and emodin on the expression of EBV lytic proteins, we assessed the expression of Rta, Zta and EA-D by indirect immunofluorescence analysis. The staining results showed that 60% P3HR1 cells express Rta, Zta and EA-D at 24 h after SB treatment (Figure 4). Meanwhile, the percentages of Rta, Zta, EA-D positive cells were gradually reduced in a dose-dependent manner after treatment with F3 and emodin, following 24 h lytic induction by SB (Figure 4). These results strengthened the evidence that F3 and emodin inhibited the expression of EBV lytic proteins induced by sodium butyrate in EBV-positive cells.

### 2.5. Quantitative Flow Cytometry Analysis of the Inhibitory Effects of F3 on EBV Lytic Proteins Expression

**Figure 5:**
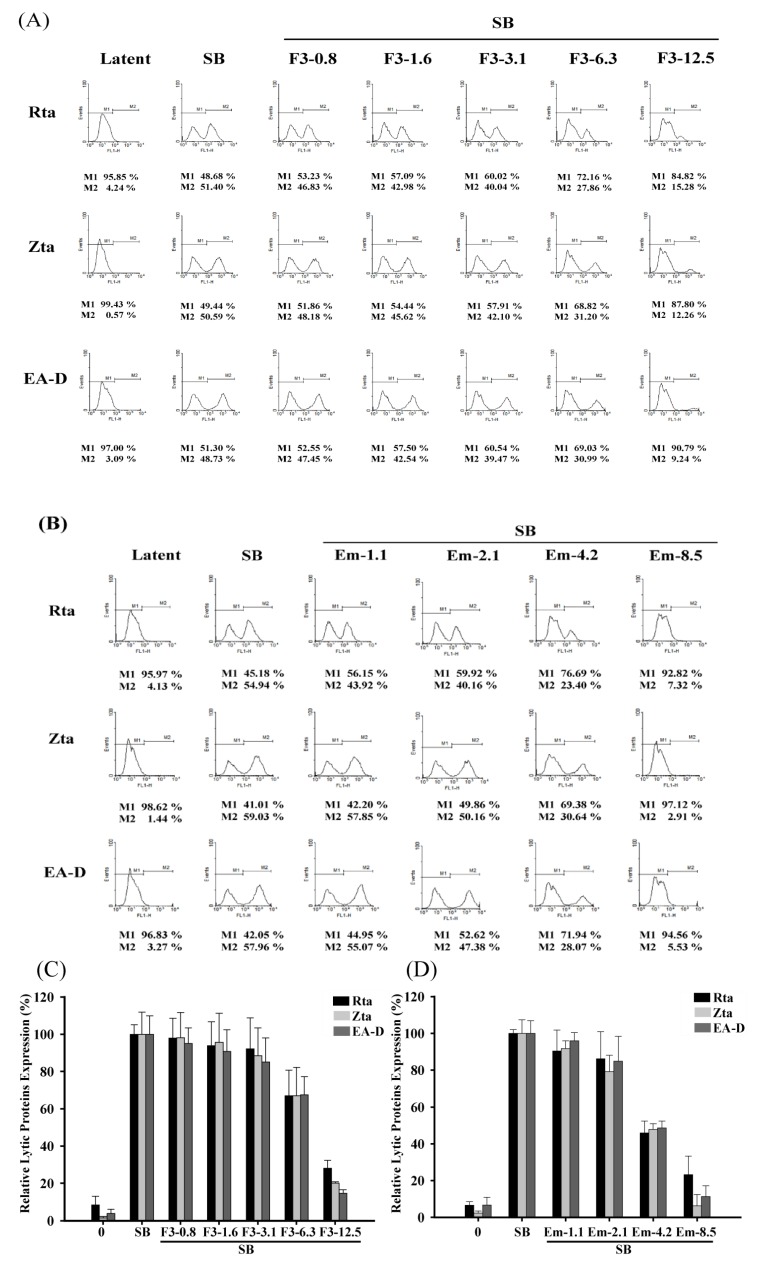
Inhibition effects of F3 and emodin on the number of cells expressing EBV lytic proteins by flow cytometric analysis. Various concentrations of F3 (0.8, 1.6, 3.1, 6.3 and 12.5 μg/mL) or emodin (Em) (1.1, 2.1, 4.2, 8.5 μg/mL) pre-treated P3HR1 cells, then SB (3 mM) co-treated cells for EBV induction. At 24 h after treatment, cells were stained with primary mouse monoclonal antibodies (anti-Rta, anti-Zta or anti-EA-D) and Alexa Fluor 488-conjugated goat anti-mouse IgG and then analyzed by flow cytometry. In histogram, M2 represented fluorescence stained cells (**A**, **B**). The number of cells expressing EBV lytic proteins was quantified by averaging the results from three independent experiments (**C**, **D**). The data were presented as means with standard deviation.

After demonstration by immunoblot and immunofluorescence analysis, we quantified the the inhibitory effects of F3 and emodin using flow cytometric analysis. The percentages of Rta, Zta, and EA-D expressing cells were estimated for determination of EBV lytic reactivation. For F3 treatment, the P3HR1 cells untreated with SB, the percentages of cells population expressing Rta, Zta and EA-D were 4.24%, 0.57% and 3.09%, respectively (Figure 5A). The population that expressed the three proteins after SB treatment increased to 51.40%, 50.59% and 48.73%, respectively. When 6.3 μg/mL of F3 was added before lytic induction, the population that expressed Rta, Zta and EA-D decreased to 27.86%, 31.20% and 30.99%, respectively (Figure 5A). The population that expressed Rta, Zta, and EA-D further decreased when the concentration of F3 increased to 12.5 μg/mL. At 12.5 μg/mL, the population that expressed Rta, Zta, and EA-D decreased to 15.28%, 12.26% and 9.24%, respectively (Figure 5A). For emodin treatment, the P3HR1 cells untreated with SB, the percentages of the cell population expressing Rta, Zta and EA-D were 4.13%, 1.44% and 3.27%, respectively (Figure 5B). The population that expressed the three proteins after SB treatment increased to 54.94%, 59.03% and 57.96%, respectively. When 4.2 μg/mL of emodin was added before lytic induction, the population that expressed Rta, Zta and EA-D decreased to 23.40%, 30.64% and 28.07%, respectively (Figure 5B). The population that expressed Rta, Zta, and EA-D further decreased when the concentration of emodin was increased to 8.5 μg/mL. At 8.5 μg/mL, the population that expressed Rta, Zta, and EA-D decreased to 7.32%, 2.91%, and 5.53%, respectively (Figure 5B). These results showed that F3 and emodin inhibited the expression of Rta, Zta and EA-D in a dose-dependent manner (Figure 5C,D). 

The concentrations of F3 required to inhibit EBV immediate-early protein expression, Rta, Zta and EA-D, by 50% (EC_50_) were 6.19, 5.9 and 5.73 μg/mL, respectively. The EC_50_s for emodin to inhibit EBV immediate-early protein expression, Rta, Zta and EA-D, were 5.2, 4.5 and 4.8 μg/mL, respectively. Although these inhibitory efficacies were inconsistent with immunoblot and immunofluorescence analyses, they provided evidence that F3 and emodin inhibited an early step of the EBV lytic cycle.

### 2.6. Inhibiting the Transcription of EBV Immediate-Early Genes

**Figure 6:**
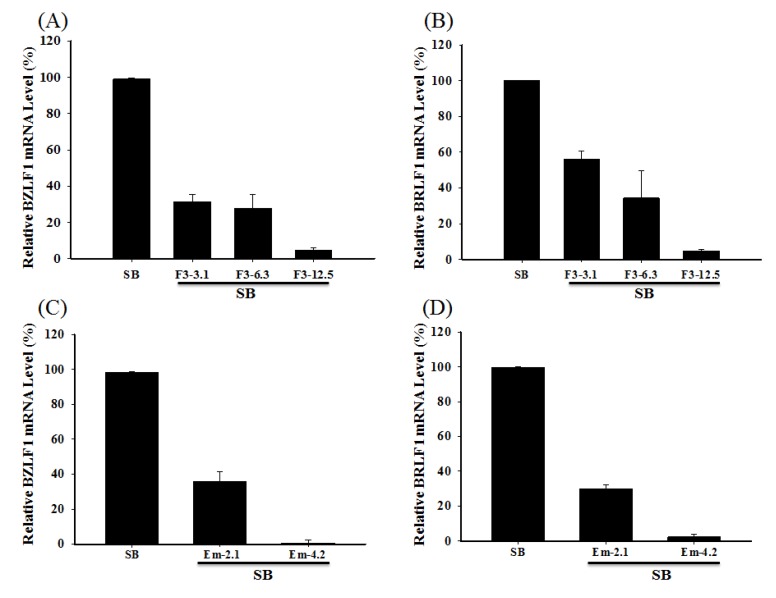
Effects of F3 and emodin on the EBV BZLF1 and BRLF1 mRNA expression**.** F3 (3.1, 6.3 and 12.5 μg/mL) or emodin (Em) (2.1 and 4.2 μg/mL) was added to P3HR1 cells before SB induction for 24 h. The expressions of BZLF1 (**A**, **C**) and BRLF1 (**B**, **D**) mRNA were detected using RT-real-time PCR.

The expressions of the BRLF1 and BZLF1 mRNA in P3HR1 cells were analyzed by RT-real-time PCR assay. The results showed that 3.1, 6.3 and 12.5 μg/mL of F3 inhibited the SB-induced BRLF1 mRNA expression by 43.87%, 65.75% and 95.29%, respectively. At these concentrations, F3 also inhibited SB-induced BZLF1 mRNA expression by 68.51%, 72.16% and 95.31%, respectively (Figure 6A). Moreover, the treatment with 2.1 μg/mL of emdoin prior to lytic induction significantly inhibited the BRLF1 and BZLF1 mRNA expression by 70.22% and 64.2%, respectively. Adding 4.2 μg/mL of emodin before lytic induction into the culture medium, entirely suppressed the BRLF1 and BZLF1 mRNA expression (Figure 6B).

### 2.7. Inhibition of the EBV DNA Replication

**Figure 7:**
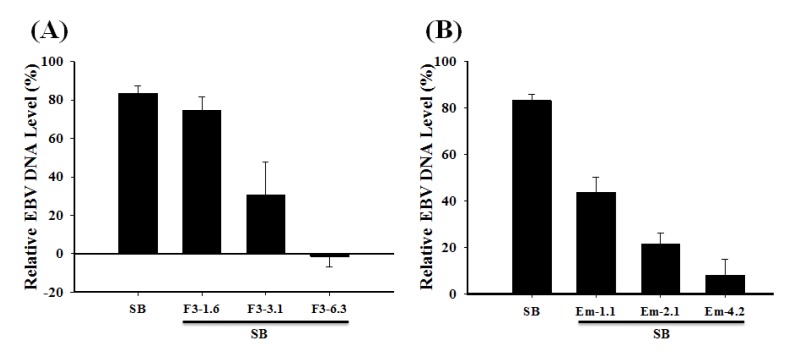
Effects of F3 and emodin on EBV DNA replication. F3 (1.6, 3.1 and 6.3 μg/mL) or emodin (Em) (1.1, 2.1 and 4.2 μg/mL) was added to P3HR1 cells before SB induction for 48 h. The expression of EBV DNA was studied by real-time PCR analysis.

P3HR1 cells were treated with 1.6–6.3 μg/mL of F3 or 1.1–4.2 μg/mL of emodin before lytic induction. After culturing for two days, EBV DNA in P3HR1 cells was isolated. Real-time qPCR was used to determine the amount of EBV DNA purified from the P3HR1 cells. These results showed that F3 and emodin at a concentration of 3.1 μg/mL and 1.1 μg/mL, respectively, decreased significantly EBV DNA replication (Figure 7). The effective concentration of F3 and emodin that inhibited EBV genome copy numbers by 50% (EC_50_) were 2.52 μg/mL and 1.2 μg/mL, respectively (Figure 7).

### 2.8. Discussion

Previous studies showed that lytic EBV proteins actually induce the expression of B-cell growth factor, IL-6, cellular IL-10 and viral IL-10, allowing B cells to grow more efficiently [25,26]. Lytically-infected cells also produce VEGF and thus contribute to angiogenesis in both B-cell and epithelial-cell malignancies [26]. Therefore, new treatment strategies aimed at completely suppressing the expression of all lytic viral proteins are useful in controlling early EBV-associated malignancies. 

In this study, we found that the ethyl acetate subfraction F3 separated from *Polygonum cuspidatum* and emodin, its main component, significantly reduce the expression of EBV immediate-early proteins, Rta, Zta and EA-D in a dose-dependent manner (Figure 3, Figure 4 and Figure 5). In other words, F3 and emodin interfere with an early step of the EBV replication cycle. The concentrations of F3 and emodin required for inhibiting EBV immediate-early proteins expression by 50% (EC_50_) are approximately 5.94 μg/mL and 4.83 μg/mL (17.87 μM), respectively. Our study also demonstrates that F3 and emodin inhibit the BRLF1 and BZLF1 mRNA expression, which in turn, affects viral lytic proteins expression. In addition, the inhibition actually decreases the EBV DNA replication. Real-time qPCR indicates that the effective concentrations of F3 and emodin that inhibit EBV genome copy numbers by 50% (EC_50_) are 2.52 μg/mL and 1.2 μg/mL, respectively (Figure 7), inconsistent with EC_50_ for inhibiting EBV immediate-early proteins expression obtained by flow cytometry (5.94 μg/mL and 4.83 μg/mL, respectively). Previous studies have showed that besides Rta and Zta proteins, the activation of EBV lytic cycle also requires other factors, such as the MBD1-containing chromatin-associated factor 1 (MCAF1) [11]. Therefore, these data implied that F3 and emodin might inhibit other lytic genes involved in EBV lytic cycle.

Earlier studies showed that epigallocatechin gallate (EGCG) [18], resveratrol [19] and andrograpolide [20] inhibit the EBV lytic cycle by suppressing the expression of the EBV immediate-early genes transcription and the expression of lytic proteins, including Rta, Zta and EA-D. The concentration of resveratrol inhibited EBV immediate-early proteins expression by 50% that obtained from flow cytometry is approximately 24 μM; EGCG inhibits the expression of EBV lytic proteins at a 50 μM concentration, which are substantially higher than the dose of emodin that effectively inhibits the EBV immediate-early proteins expression by 50%, 17.87 μM. Moreover, andrographolide effectively inhibits the EBV lytic cycle at 14 μM, while 1.2 μg/mL (4.4 μM) of emodin inhibits EBV DNA replication by 50%, suggesting that emodin is more effective than EGCG, resveratrol and andrograpolide in inhibiting EBV reactivation.

The molecular mechanism underlying the inhibition of EBV early gene expression by the ethyl acetate subfraction F3 separated from *Polygonum cuspidatum* is unclear. Results showed that the ethyl acetate subfraction F3 contains 68.2% of emodin (Figure 1). Comparison of the EC_50_ values of F3 and emodin that inhibit the expression of EBV immediate-early proteins and DNA replication (5.94 μg/mL and 4.83 μg/mL, 2.52 μg/mL and 1.2 μg/mL, respectively), suggests that emodin is a major bioactive compound responsible for the EBV lytic cycle suppressing activity of the ethyl acetate subfraction F3 separated from *Polygonum cuspidatum*. Our previous studies showed that the ethanolic extract of *P. cuspidatum* (PcE) inhibits the EBV lytic cycle [24]. Results showed that PcE contains 5% of emodin and the concentration of PcE required to inhibit EBV immediate-early protein expression by 50% is approximately 29 μg/mL. Moreover, this study showed the concentration of emodin required for inhibiting EBV immediate-early proteins expression by 50% is approximately 4.83 μg/mL, implying that in addition to emodin other bioactive compounds such as resveratrol may also be involved in the PcE suppression of EBV immediate-early proteins [19]. Previous studies have reported that emodin inhibits the activation of p38 MAPK, ERK and JNK signaling [27] and affects the activation of the promoters that are activated by AP-1 [28] and ATF2 [29], as both the BRLF1 and BZLF1 promoters are strongly activated by AP-1 and ATF1 [30]. Therefore, the inhibition of emodin on activation of signaling pathways may be involved in the inhibition of F3 and emodin on the EBV lytic cycle. 

## 3. Experimental

### 3.1. Material

*P. cuspidatum* was collected from the San-Dei-Men area in Pingtung County, Taiwan and verified by Prof. C. S. Kuoh. The specimen was deposited in the herbarium of the National Cheng Kung University, Tainan, Taiwan. Emodin was purchased from Sigma Chemical Co. (St. Louis, MO, USA).

### 3.2. Sample Preparation

Ten grams of dried powder from the *P. cuspidatum* root was extracted three times in 100 mL of ethanol by refluxing at 85 °C for 2 h. After each extraction, the ethanol fraction was collected by filtration. The ethanol fractions were partitioned twice with hexane and water (10:10:1). The aqueous fractions were collected, concentrated under reduced pressure, and called PcE(H). Dried PcE(H) (1 g) dissolved in 100 mL distilled water was partitioned with an equal volume of ethyl acetate (EtOAc). The EtOAc fractions were collected, concentrated under reduced pressure, and called PcE(H)E. The PcE(H)E soluble fraction was subjected to semi-preparative HPLC using a HYPERPREP C18 (150 × 10 mm i.d., 8 μm) column (Thermo Fisher, Waltham, MA, USA) and eluted with a mobile phase consisting of (A) water and (B) methanol. Gradient elution was performed as follows: 30%–50% B in 0–15 min, 50%–90% B in 15–35 min, and 95% B in 35–45 min. An ethyl acetate subfraction F3 and a yield of 0.31% were obtained. The resulting residues were finally dissolved in dimethyl sulfoxide (DMSO).

### 3.3. HPLC Analysis

The components in the ethyl acetate subfraction F3 were analyzed by high performance liquid chromatography (HPLC) using a LiChrospher 100 RP-18e (250 × 4.6 mm i.d., 5 μm) column (Merck, Darmstadt, Germany). The mobile phase consisted of (A) water and (B) methanol. Gradient elution was performed as follows: 30%–50% B in 0–15 min, 50%–90% B in 15–35 min, and 95% B in 35–45 min. The flow rate was set at 1 mL/min. The effluent was monitored from 210 to 500 nm using a diode array detector.

### 3.4. Cell Culture and Lytic Induction of EBV

P3HR1, a Burkitt’s lymphoma cell line that was latently infected by EBV, was cultured in RPMI 1640 medium containing 10% fetal calf serum (Biological Industries, Kibbutz Beit HaEmek, Israel). Cells were treated with 3 mM of sodium butyrate (SB) to induce the EBV lytic cycle [31]. 

### 3.5. Cell Viability Assay

A 1 mg/mL solution of [3-(4,5-dimethyldiazol-2-yl)-2,5-diphenyltetrazolium bromide (MTT) in RPMI 1640 medium was added into 1 × 10^5^ P3HR1 cells. The dehydrogenase activity of the viable cells was measured using the method of Carmichael *et al.* [32].

### 3.6. Immunoblot Analysis

Cell lysate was prepared from 3 × 10^6^ P3HR1 cells with 100 μL of lysis buffer that contained 50 mM Tris-HCl, pH 7.8, 150 mM NaCl, 5 mM EDTA, 0.5% Triton X-100 and 0.5% NP40 using a method described elsewhere [18]. SDS-polyacrylamide gel electrophoresis and immunoblot analysis with anti-Rta, anti-Zta and anti-EA-D antibodies, which were purchased from Argene (Varilhes, France), were performed using methods described previously [33].

### 3.7. Indirect Immunofluorescence Staining

P3HR1 cells were plated on poly-L-lysine-coated overslips and fixed with 4% paraformaldehyde at 4 °C for 15 min, followed by treatment with PBS containing with 0.5% Triton X-100 for 10 min at room temperature. After blocking with 1% BSA in PBS at room temperature for 30 min, monoclonal anti-Rta, anti-Zta, and anti-EA-D antibodies were applied at a dilution of 1:200 and 1:1000, respectively and incubated at room temperature for 1 h. Next, the cells were washed with PBS and incubated with the Alexa Fluor 488-conjugated goat anti-mouse IgG for 1 h. DNA was visualized by staining with 4',6'-diamidio-2-phenylindole (DAPI). Cells were examined under the Axioskop 2 plus fluorescence microscope (Zeiss, Thornwood, NY, USA).

### 3.8. Flow Cytometric Analysis

To determine the number of cells expressing EBV lytic proteins, cells were treated and harvested, fixed in 4% paraformaldehyde. The fixed cells were permeabilized with 0.5% Triton X-100 in PBS and incubated with mouse monoclonal anti-Rta (dilution 1:250), anti-Zta (dilution 1:250) (Argene), and anti-EA-D antibodies (dilution 1:2500) (Chemicon, Temecula, CA, USA). Secondary antibodies used in the study included Alexa Fluor 488-conjugated goat anti-mouse IgG from Invitrogen (Carlsbad, CA, USA). Finally, the cells were resuspended in 1% paraformaldehyde and analyzed using a flow cytometer (Model FACScanTO, BD Biosciences, San Jose, CA, USA).

### 3.9. RNA and DNA Extraction

For the real-time quantitative PCR assay, RNA was extracted from 3 × 10^6^ cells using a conventional Trizol-chloroform method (Invitrogen). RNase-Free DNase Set (Promega, Madison, WI) performed DNA removal. Total RNA was added to High capacity cDNA reverse transcription kit (Applied Biosystems, Foster, CA, USA) to produce cDNA. Briefly, 10 μL of treated RNA (2 μg) was mixed with 0.8 μL 25 × dNTP mix (100 mM) and 2 μL 10 × RT Random Primers, followed by adding 2 mL 10 × RT buffer, 4.2 μL 0.1% DEPC water and 1 μL MultiScribe™ Reverse Transcriptase (50 U/μL). The components in tube were mixed gently and incubated for 10 min at 25 °C, for 120 min at 37 °C and 5 min at 85 °C. DNA was extracted using Quick-gDNA miniPrep kit (Zymo Research, Irvine, CA, USA). 

### 3.10. Real-Time Quantitative PCR

For mRNA analysis, quantitative PCR (qPCR) was performed with the use of SYBR green (Applied Biosystems), for DNA analysis was performed with Taqman probe (5'-6-FAM-GGAGACACATCTGGACCAG-MGBNFQ-3') on an ABI StepOne™ real-time PCR system with StepOne™ software v 2.1. All reactions were run in triplicate. Mean cycle threshold (C_T_) values were normalized to β-actin, yielding a normalized C_T_ (ΔC_T_). The ΔΔC_T_ value was calculated by subtracting respective control from the ΔC_T_, and expression level was then calculated by 2 raised to the power of the respective −ΔΔC_T_ value. Relative mRNA (DNA) level (%) = 2^−ΔΔCT^ (SB and F3 treatment)/2^−ΔΔCT^ (SB treatment) × 100. Primers for mRNA analysis include the following: BRLF1 forward (5'-TCACTACACAAACAGACGCAGCCA-3') and reverse (5'-AATCTCCACACTCCCGGCTGTAA-3'); BZLF1 forward (5'-AGAAGCACCTCAACCTGGAGACAA-3') and reverse (5'-CAGCGATTCTG GCTGTTGTGGTTT-3'); and β-actin forward (5'-CGTCTTCCCCTCCATCG) and reverse (5'-CTCG TTAATGTCACGCAC-3'). Primers for DNA analysis, EBNA1 forward (5'-TACAAGACCTGGAAA GGCC-3') and reverse (5'-TCTTTGAGGTCCACTGCC -3').

### 3.11. Statistical Analysis

Data were analyzed statistically by one-way analysis of variance (ANOVA) followed by Dunnett’s *post hoc* test using the SAS JMP 8.0 software package. Values are expressed as mean ± SD of three replicates and a *p* value of <0.05 was regarded as significant.

## 4. Conclusions

The results of this study clearly demonstrated that F3 and emodin inhibit the transcription of lytic genes and the lytic cycle of EBV to reduce viral DNA replication. Thus, F3 and emodin could potentially be used for the development of anti-EBV drugs.
